# The prevalence of asthma in adult population of southwestern Iran and its association with chronic rhinosinusitis: a GA^2^LEN study

**DOI:** 10.1186/s13601-019-0283-6

**Published:** 2019-08-30

**Authors:** Afshin Ostovar, Wytske J. Fokkens, Safoora Pordel, Ali Movahed, Khadijeh Ghasemi, Maryam Marzban, Shokrollah Farrokhi

**Affiliations:** 1 Osteoporosis Research Center, Endocrinology and Metabolism Clinical Sciences Institute, Tehran University of Medical Sciences, Tehran, Iran; 2 Department of Otorhinolaryngology, Academic Medical Center, Amsterdam, The Netherlands; 3 Department of Immunology and Allergy, The Persian Gulf Tropical Medicine Research Center, The Persian Gulf Biomedical Research Institute, Bushehr University of Medical Sciences, Sangi St, PO Box: 75 16 68 88 76, Bushehr, Iran; 4 The Persian Gulf Tropical Research Center, Biochemistry Group, Bushehr University of Medical Sciences, Bushehr, Iran; 5 Department of Pediatrics, Faculty of Medicine, Bushehr University of Medical Sciences, Bushehr, Iran; 6 Department of Epidemiology, School of Public Health, Bushehr University of Medical Sciences, Bushehr, Iran

**Keywords:** Asthma, Chronic rhino sinusitis, GA^2^LEN, Prevalence

## Abstract

**Background:**

Asthma is recognized as a major public health concern in the world. The aim of this investigation was to evaluate the prevalence of asthma by using the Global Allergy and Asthma Network of Excellence (GA^2^LEN) questionnaire and examine its association with chronic rhinosinusitis, in the province of Bushehr, Southwestern of Iran.

**Methods:**

In a cross-sectional, population-based study, a total of 5420 invited individuals, aged 15–65, were selected through a multi-stage, stratified, cluster random sampling and from which 5201 completed the GA^2^LEN questionnaire (response rate = 95.9%). The prevalence of asthma, current, and physician-diagnosed asthma were analyzed by using sex and age groups and the association of asthma and chronic rhinosinusitis (CRS) was investigated using a multiple logistic regression model.

**Results:**

Based on the information from the GA^2^LEN questionnaire, the overall prevalence of asthma in the population under study was 10.0% (95% CI 9.2–10.8). Moreover, the prevalence of current asthma was 8.9% (95% CI 8.1–9.7). Further, the prevalence of current early, late-onset and physician-diagnosed asthma within the asthma group was 51.1% (95% CI 46.5–55.7), 48.9% (95% CI 44.3–53.5) and 3.9% (95% CI 2.1–2.5), respectively. Additionally, CRS was more frequent among the participants with asthma [(57.3%, OR = 2.3; 95% CI 2.1–2.5)], and there was a significant association between CRS and current, early and late-onset of asthma (P < 0.001; OR = 4.4, 3.2 and 6, respectively).

**Conclusion:**

This large population study conducted in the southwestern part of Iran suggests that the prevalence of asthma is high. Moreover, the result of this study showed a strong association of asthma with CRS; also after adjusting for sex, age, educational level, and smoking.

## Introduction

Asthma is one of the most common respiratory disease affecting about 330 million people throughout the world [1]. It is characterized by chronic inflammation, airflow obstruction and airway hyperresponsiveness [2]. The symptoms include episodes of wheezing, coughing, chest tightness, and shortness of breath [3]. In the development of allergic asthma, both genetic predisposition and environmental factors like exposure to air pollution and allergens play a role [4, 5]. The disease is associated with poor quality of life, considerable healthcare costs and loss of work productivity [6–9]. The global prevalence of asthma in adults reported being 4.3% with wide variation among different countries [1, 10]. Reports from the standardized studies have shown the prevalence of the disease to varying from 1 to 18% in the general population of different countries [11–13]. Furthermore, asthma is more prevalent in developed countries comparing to the developing world and a study was conducted in the adult population of Tehran, Iran in 2017 using European Community Respiratory Health Survey (ECRHS) questionnaire, showed the prevalence of asthma as 8.9% (95% CI 8.5–9.3) [14]. Although there are a few systematic reviews about the prevalence of childhood asthma in Iran, we could not find any systematic review and meta-analysis on the prevalence of asthma in the adult population of Iran. Based on the results of a meta-analysis in the childhood population, prevalence of a history of asthma and current asthma was 4.36% and 8.8%. In addition, the prevalence of asthma was varied at 5.7–10.5% between 1996 and 2014 in Iran [15]. Importantly, a number of conditions are associated with asthma including chronic rhinosinusitis (CRS). Therefore, studies revealed that over 40% of the patients diagnosed with asthma had CRS and 50% of the patients with CRS, especially those with a nasal polyp, had asthma [16]. Population-based studies have provided critical data for the assessment of chronic diseases related to the respiratory system for the management of health policies [17]. The Global Allergy and Asthma European Network (GA^2^LEN), the Sixth EU Framework Program for Research and Technological Development (FP6) Network of Excellence, proposed a standardized questionnaire for the assessment of the prevalence of respiratory allergic diseases [18]. As Iran is a country of variable population and climate, it can be expected that the prevalence of asthma varies in different regions. Since Bushehr port is located in the southwestern part of Iran with a high temperature and humidity which sometimes reaches 90%. The vegetation mapping of the region is mainly palm dates having yearly pollination in the spring season. Moreover, Bushehr nuclear power plant is located close to the city and unlike the situation in Europe; it is not an industrial region. Most of the time, people living in the area are exposed to the sea-originated moisture and allergens, pollens from palm trees as well as the dusty air pollution. Therefore, we designed the present study to assess the prevalence of asthma and its association with CRS in adults’ population in Bushehr with respect to the GA^2^LEN questionnaire as a standard method.

## Materials and methods

### Study design and sampling

**Fig. 1:**
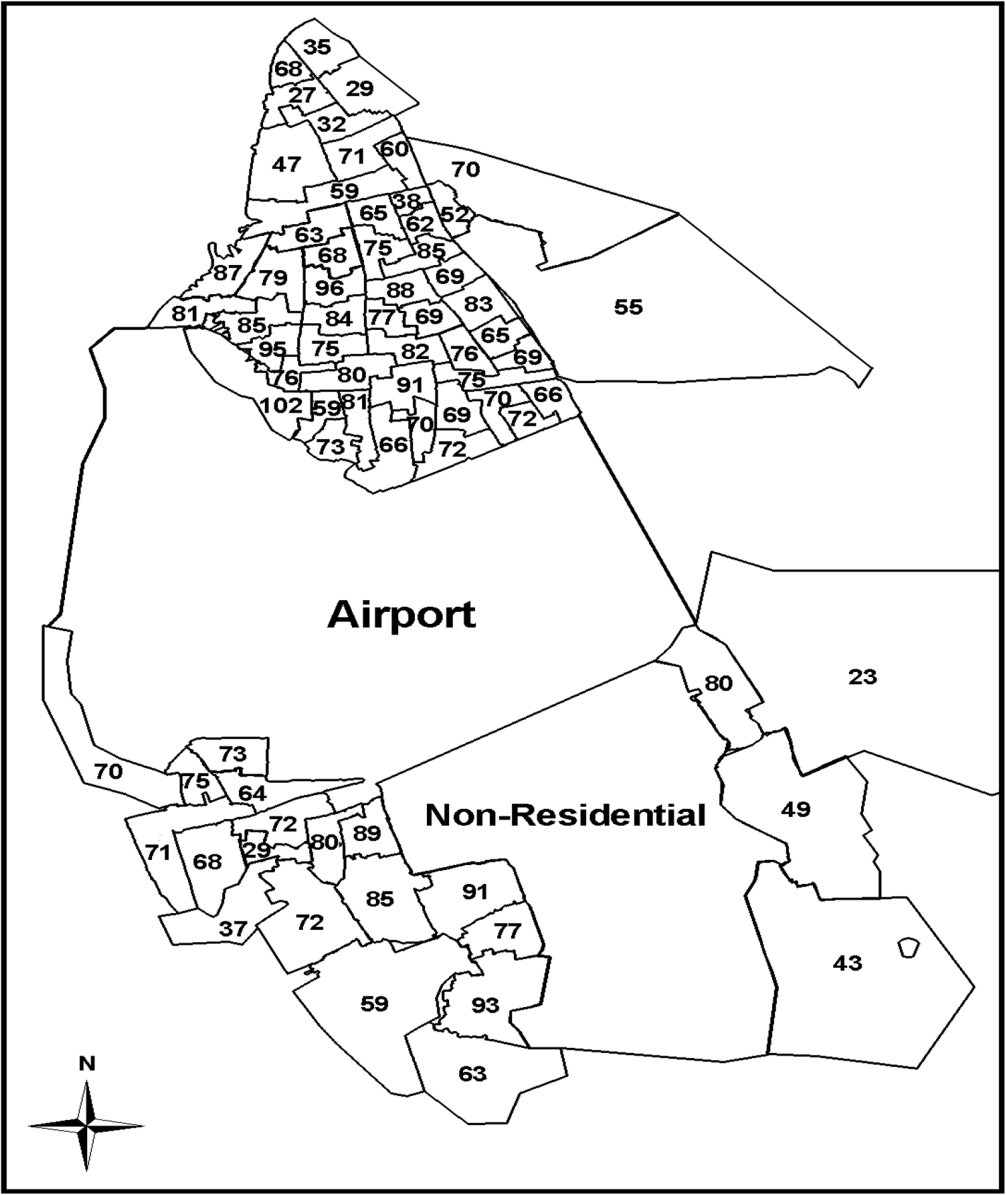
Map of Bushehr and distribution of participants in the strata

In a cross-sectional population-based study, 5420 participants aged between 15 and 65 years, were chosen and stratified randomly through a multistage cluster sampling. The inclusion criteria were the participants must have been residents of the city of Bushehr for at least 1 year. According to the classification given by the governing municipality, as shown in Fig. 1, the city of Bushehr was stratified into 75 strata, and numbers were assigned to the different blocks (cluster type) of each stratum and then they were randomly sorted. Therefore, the sample size for strata was determined according to the number of households residing in each stratum. Finally, the data from 5201 participants were collected and analyzed. Therefore, the response rate in our study was 95%. The participants were interviewed in person by an experienced researcher and face to face at their doors. Moreover, a standardized questionnaire was delivered to them to be completed and collected by the investigator the next day.

The present study was approved by the Ethics Committee of Bushehr University of Medical Sciences, Bushehr, Iran (Ethical approval code: IR.BUMPS.REC.1395.123-2).

### Questionnaire and data collection

A Persian translation of the questionnaire (GA^2^LEN) was used which included the questions about the socioeconomic and educational status of the participants [19].

### Definitions

Different educational levels were defined as having no education, school (primary, secondary and higher) and university levels. The final questionnaire was checked for face-validity of questions. Current asthma defined as a positive answer to the question “Have you ever had asthma in the last 12 months?” and at least having one of 3 symptoms: wheezing or whistling in the chest, waking with breathlessness or an asthma attack. In addition, diagnosed asthma was defined as a positive answer to the questions “Have you ever had asthma?” and “Are you taking any medication or administration for asthma?” The other primary outcomes were defined as current late-onset asthma (reported to have started at or after the age of 16) and current early-onset asthma (reported to have an onset before 16 years of age). Asthma attack was also defined as two questions: “Have you ever been hospitalized with asthma?” and “Have you had an attack of asthma in the last 12 months?”. We defined CRS based on the European Position Paper on Rhinosinusitis and Nasal Polyps (EPOS) criteria 2012 for epidemiological studies [20].

### Statistical analysis

The prevalence of asthma was calculated to be as 95% confidence intervals using the exact binomial method. The prevalence of asthma was compared by age group (15–24, 25–34, 35–44, 45–54, 55–65), gender (male or female), smoking status (cigarette/water pipe smoker or current smoker), and education levels using Chi-Square Tests. Crude and adjusted odds ratios were estimated using multiple logistic regression models, to investigate the association of asthma and CRS. Statistical analyses were performed using SPSS 21 software package (San Diego, CA) and the P values of < 0.05 was considered statistically significant.

## Results

**Fig. 2:**
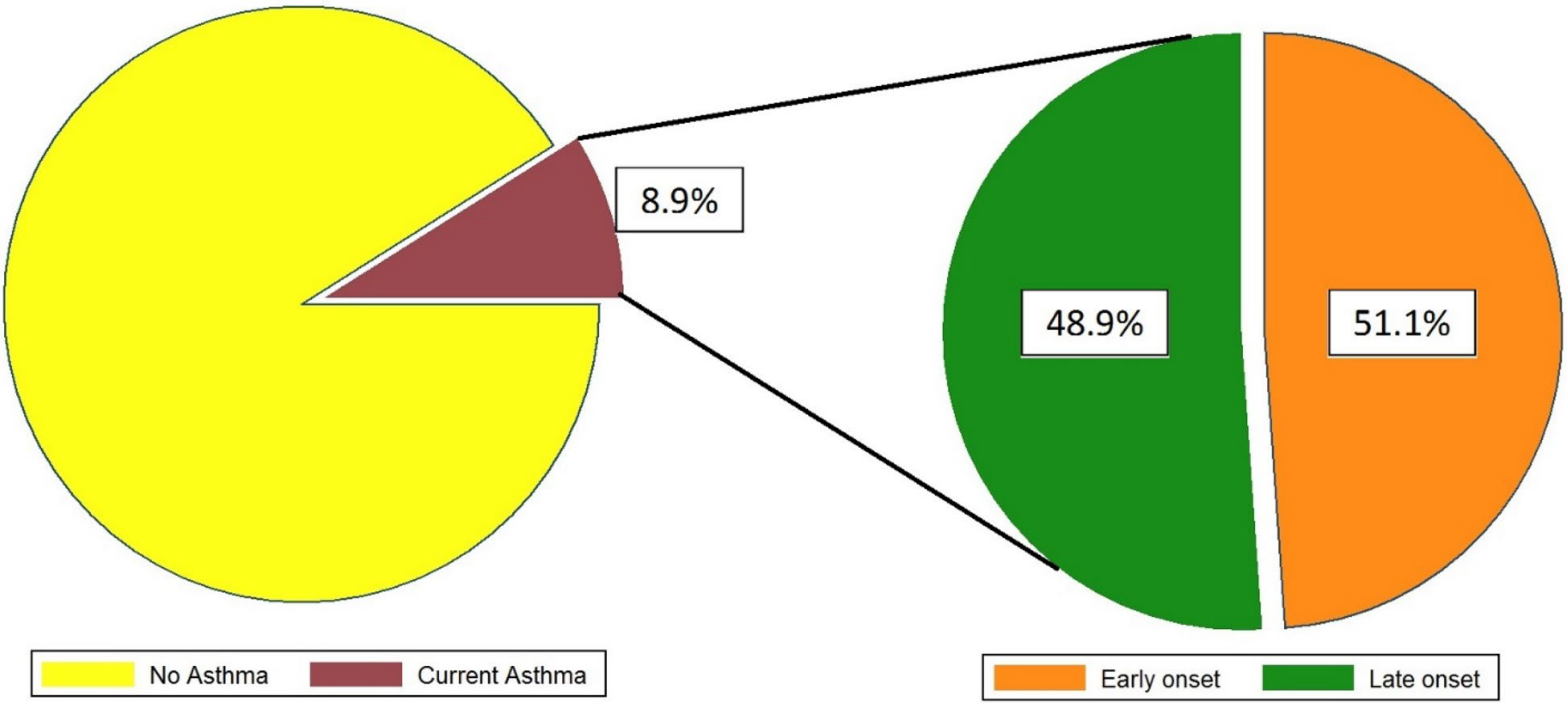
The total prevalence of current asthma and the prevalence of current early and late-onset asthma in patients with current asthma

The socio-demographic data of the participants are shown in Table 1. Based on the results of this study, the total prevalence of asthma and the current asthma was 10.0% (95% CI 9.2–10.8) and 8.9% (95% CI 8.1–9.7), respectively. Of the total individuals with asthma, the prevalence of current early and late-onset asthma was 51.1% (95% CI 46.5–55.7) and 48.9% (95% CI 44.3–53.5), respectively (Fig. 2). Moreover, the prevalence of physician-diagnosed asthma was 3.9% (95% CI 2.1–2.5).

**Table 1:** Socio-demographic characteristics of the participants

Variation	Variation subgroup	Asthma (n = 502)	No asthma (n = 4536)	P value*
Sex	Female	282 (56.1)	2315 (51)	0.050
	Male	220 (43.9)	2221 (49)	
Age group	15–24	100 (21.5)	1070 (24.8)	
	25–34	109 (23.4)	1050 (24.3)	0.558
	35–44	100 (21.5)	900 (2.86)	
	45–54	92 (19.8)	771 (17.9)	
	≥ 55	64 (13.8)	524 (12.1)	
Education level	No education	16 (15.4)	88 (2.0)	*<* *0.001*
	Primary school	39 (14.6)	229 (5.2)	
	Secondary school	77 (12.1)	558 (12.8)	
	High school	139 (9)	1408 (32.2)	
	University	204 (8.9)	2086 (47.8)	
Smoking		83 (16.8)	423 (9.4)	*<* *0.001*
Health workers		12 (2.6)	73 (1.7)	0.154
Cleaning worker		26 (5.9)	111 (2.7)	*0.001*

Italic values indicate significant of P value (P < 0.05)

* P values for Pearson Chi-square test

Of the total participants reported having asthma, 238 (47.5%) had an asthma attack during the last 12 months. The frequency of symptoms in patients having an asthma attack in comparison with those without attack was shown in Table 2. Furthermore, those who had asthma for the last year had more hospitalization as compared to the others (54% vs. 30%) (OR = 2.7; 95% CI 1.87–4.03).

**Table 2:** The frequency of symptoms in patients with and without asthma attack

Symptom	Asthmatic patients with asthma attack in the last year N = 238 (47.5%)	Asthmatic patients without asthma attack in the last year N = 264 (52.5%)	P-value
Wheeze	175 (60%)	117 (40%)	*<* *0.001*
Chest tightness	146 (61%)	60 (39%)	*<* *0.001*
Woken by shortness of breath	143 (60%)	96 (40%)	*<* *0.001*
Woken by coughing	174 (55%)	140 (44.5%)	*<* *0.001*
Phlegm	169 (56%)	131 (44%)	*<* *0.001*

Italic values indicate significant of P value (P < 0.05)

The present study showed that the prevalence of CRS was higher among the participants with asthma (57.3%) than those without asthma (24.6%) (OR = 2.3; 95% CI 2.1–2.5). Finally, there was a significant association between CRS and the current, early and late-onset of asthma (P < 0.001; OR = 4.4, 3.2 and 6, respectively) after adjusting for sex, age, educational level, and smoking (Table 3).

**Table 3:** The association between chronic rhinosinusitis and asthma

	CRS N = 1362 (27.8)	No CRS N = 3530 (72.1)	Crude OR^a^	P-value	Adjusted OR^b^	P-value
Current asthma	223 (16.3)	154 (4.3)	4.2 (3.4 to 5.3)	*0.001*	4.0 (3.2 to 5.1)	*0.001*
Current early-onset asthma	86 (6.3)	87 (2.4)	2.9 (2.1 to 4.0)	*0.001*	2.8 (2.0 to 3.9)	*0.001*
Current late-onset asthma	137 (10.0)	67 (1.8)	6.11 (4.5 to 8.2)	*0.001*	5.6 (4.1 to 7.7)	*0.001*
Physician-diagnosed asthma	86 (6.3)	42 (1.1)	6.2 (4.3 to 9.1)	*0.001*	5.3 (3.6 to 7.8)	*0.001*

Italic values indicate significant of P value (P < 0.05)

^a^ORs were provided using Multi Variable Logistic Regression

^b^Adjusted for sex, age, education level

## Discussion

Asthma is the most common chronic respiratory disease with high prevalence throughout the world. The disease is known to be caused by a combination of genetic and environmental factors, including changes in lifestyle, urbanization and mainly air pollution [21]. In the present study, we evaluated the prevalence of various epidemiological types of asthma, including diagnosed and current asthma, based on the data obtained from the GA^2^LEN standard questionnaire. The difference in the response rate (95%) in our study may be because of the way to approach the participants at their house by an experienced investigator, especially face to face; and by explaining everything to the individuals before getting all the information needed. Our results showed the prevalence of asthma in the area under study to be 10%, which is slightly higher than the 8.9% found in Tehran, Iran [14]. Furthermore, it was found that the prevalence of asthma in Bushehr was high as compared to other regions in Iran. Additionally, the prevalence of current asthma estimated to be 8.9% in the present study which was in agreement with that of current asthma (8.8%) already reported by a meta-analysis in Iran [15]. Moreover, the prevalence of physician-diagnosed asthma was 3.9% in our study which was higher than the result obtained by the meta-analysis (2%) [15]. In Europe, studies using the GA^2^LEN questionnaire resulted in the prevalence of current asthma within a small range of 6.0–7.6% [8, 17]. On the contrary to the reports given by Song et al. in 9 Asian countries showed an extensive variation (0.7% to 11.9%) in the prevalence of adult asthma [22]. In our previous study which was conducted in 2014 using the International Study of Asthma and Allergies in Childhood (ISAAC) questionnaire for childhood, the prevalence of asthma in children living in Bushehr was found to be 6.7% (6–7-year-olds) and 7.6% (13–14 year-olds) [23].

Furthermore, the results of the present study showed that the prevalence of asthma was more in younger individuals (25–34 years old) whose atopy occurred within the same range [24]. But, the prevalence of asthma in the young population of some countries has been reported to be less as compared to our result [25]. In addition, the present study showed that the prevalence of allergic rhinitis was 28.8%. Meanwhile, the prevalence of allergic rhinitis in children with 13–14 year- old was reported to be high (30%) in Bushehr [23]. Therefore, a higher prevalence of asthma may be related to the higher occurrence of allergic rhinitis in the region.

Additionally, subjects with lower educational level had a higher prevalence of asthma which is in line with other studies [14, 26].

To our knowledge, this was the first population-based study that demonstrated the association between asthma and CRS (based on EPOS criteria) in Iran. Moreover, we found that 57.3% of the individuals with the symptoms of asthma reported having CRS and there was an association between asthma and CRS (OR: 2.3) after adjusting for sex, age, educational standard level, and smoking. Further, epidemiological studies reported that asthma and CRS are strongly associated; studies reported that 40% of asthmatic patients may have CRS, and they are caused by the chronic inflammation of united airways [12, 27–30]. Moreover, the production of inflammatory mediators and cytokines and their subsequent actions at the upper and lower levels of the respiratory tract, confirms the relationship between asthma and CRS [31]. Therefore, they are coexisting in the airway as a single disease [32]. Importantly, our data revealed that CRS was positively associated with current, early and late-onset of asthma. The European study has reported that CRS was associated positively with adult-onset and negatively with childhood-onset of asthma [12]. Moreover, another study has reported a strong relationship between the increase in severity of asthma and CRS in adults; the result of the study also showed that the severity of asthma may have a significant relation with the presentation of CRS [33, 34].

## Conclusion

The results of the present study show that the prevalence of asthma in adults living in southwestern Iran was high. And a strong association was found between asthma and CRS, after adjusting for sex, age, educational status, and smoking.

## Data Availability

The datasets used and analysed during the current study are available from the corresponding author on reasonable request.

## Abbreviations

GA^2^LEN: Global Allergy and Asthma European Network
CRS: chronic rhinosinusitis
EPOS: European Position Paper on Rhinosinusitis and Nasal Polyps
ECRHS: European Community Respiratory Health Survey
ISAAC: International Study of Asthma and Allergies in Childhood
